# Rapid HPLC method reveals dynamic shifts in coenzyme Q redox state

**DOI:** 10.1016/j.jbc.2024.107301

**Published:** 2024-04-17

**Authors:** Victor Vitvitsky, Roshan Kumar, Jutta Diessl, David A. Hanna, Ruma Banerjee

**Affiliations:** 1 Department of Biological Chemistry, Michigan Medicine, University of Michigan, Ann Arbor, Michigan, USA; 2 Center for Theoretical Problems of Physico-Chemical Pharmacology, Russian Academy of Sciences, Moscow, Russia

**Keywords:** coenzyme Q, redox, electron transport chain, hypoxia, hydrogen sulfide

## Abstract

Ubiquinol or coenzyme Q (CoQ) is a lipid-soluble electron carrier in the respiratory chain and an electron acceptor for various enzymes in metabolic pathways that intersect at this cofactor hub in the mitochondrial inner membrane. The reduced form of CoQ is an antioxidant, which protects against lipid peroxidation. In this study, we have optimized a UV-detected HPLC method for CoQ analysis from biological materials, which involves a rapid single-step extraction into *n*-propanol followed by direct sample injection onto a column. Using this method, we have measured the oxidized, reduced, and total CoQ pools and monitored shifts in the CoQ redox status in response to cell culture conditions and bioenergetic perturbations. We find that hypoxia or sulfide exposure induces a reductive shift in the intracellular CoQ pool. The effect of hypoxia is, however, rapidly reversed by exposure to ambient air. Interventions at different loci in the electron transport chain can induce sizeable redox shifts in the oxidative or reductive direction, depending on whether they are up- or downstream of complex III. We have also used this method to confirm that CoQ levels are higher and more reduced in murine heart *versus* brain. In summary, the availability of a convenient HPLC-based method described herein will facilitate studies on CoQ redox dynamics in response to environmental, nutritional, and endogenous alterations.

A relatively late addition to the legion of known cellular redox cofactors, ubiquinone or coenzyme Q (CoQ)‡ was discovered in the mid-to-late 1950’s and identified soon after as an intermediate carrier in the electron transport chain (ETC) (1, 2). The 1,4-benzoquinone head group of CoQ is its redox active moiety and undergoes reversible one- or two-electron reduction to the semiquinone and ubiquinol, respectively (Fig. 1*A*) (3). The hydrophobic tail, comprising 9 (CoQ_9_) or ten (CoQ_10_) repeats of the 5-carbon isoprenoid unit, represents the dominant forms of the cofactor found in mammals. CoQ harvests electrons from two major ETC complexes, NADH:ubiquinone oxidoreductase (complex I) and succinate dehydrogenase (complex II), and funnels them to ubiquinol:cytochrome *c* oxidoreductase (complex III) (Fig. 1*B*). CoQ also accepts electrons from a number of other enzymes, including sulfide quinone oxidoreductase (SQOR), proline dehydrogenase, dihydroorotate dehydrogenase, choline dehydrogenase, glycerol-3-phosphate dehydrogenase and the electron transfer flavoprotein-ubiquinone oxidoreductase, and is thus at the crossroads of diverse metabolic pathways. The availability of the oxidized cofactor can impact multiple cellular reactions, while its redox state influences the cellular antioxidant capacity as well as the direction of electron flow in the ETC (4). A reductive shift in the CoQ pool can lead to reverse electron transfer through complex I (5), promote the use of fumarate as a terminal electron acceptor by complex II (6, 7), and/or increase reactive oxygen species production, with oxidative and stress signaling implications (8).

**Figure 1:**
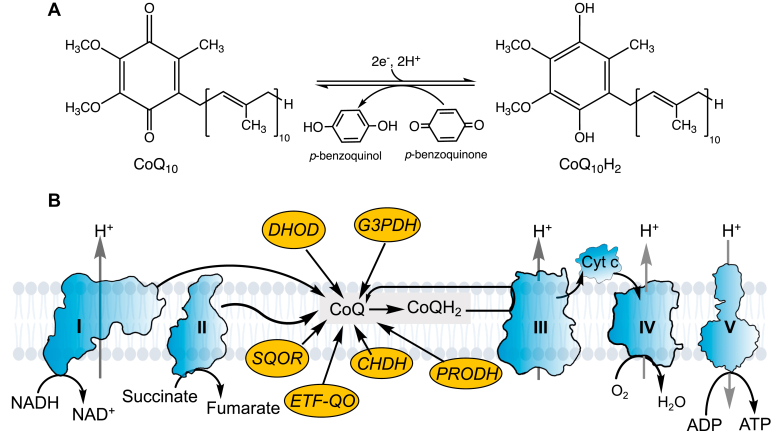
**CoQ structure and its role in the ETC.***A*, structures of CoQ_10_ and CoQ_10_H_2_ and oxidation of the latter with *p*-benzoquinone, which is used in this study. *B*, CoQ_10_ is a central electron acceptor not only in the ETC but also for a host of other mitochondrial enzymes including sulfide quinone oxidoreductase (SQOR), electron transfer flavoprotein ubiquinol oxidoreductase (ETF-QO), choline dehydrogenase (CHDH), proline dehydrogenase (PRODH), glycerol 3-phosphate dehydrogenase (G3PDH), and dihydroorotate dehydrogenase (DHOD). I-IV denotes complexes I-IV, complex V is ATP synthase and Cyt c is cytochrome c.

In addition to the mitochondrion, which is estimated to house a sizeable proportion of the cellular CoQ pool (9), the cofactor is found in the plasma membrane and in endo-membranes. For example, in the plasma membrane, the NADPH-dependent ubiquinone oxidoreductase ferroptosis suppressor protein FSP1 plays an important role in protecting lipids against peroxidation and suppressing ferroptosis (10). The mechanisms by which CoQ, which is synthesized in the mitochondrion, is distributed to other membranes, and how this process is regulated, are not understood (11).

While ubiquinol with varying isoprenoid tail lengths is the dominant quinone in mammals, a variety of alternative quinones are found in microbes, including menaquinone, naphthoquinone, and rhodoquinone, which serve not only as electron carriers in canonical ETCs, but also support bidirectional extracellular electron transfer to/from minerals, regulate gene expression, and influence colonization and virulence (12, 13). Much remains to be learned about the diverse structures, uses, and regulation of bacterial quinones and the interactions between host and microbial quinone pools.

HPLC-based separation coupled to UV (14, 15, 16) or electrochemical (17, 18) detection is said to be the “gold standard” for analysis of total ubiquinone levels and its redox status, respectively (19). More recently, liquid chromatography–mass spectrometry-based methods have been developed, which allow simultaneous monitoring of the reduced and oxidized cofactor pools (20, 21). Susceptibility of ubiquinol to oxidation, differences in sample handling procedures, and the use of variable standards have contributed to a range of reduced:oxidized CoQ values reported for cells, tissues, food, and clinical samples (19).

In this study, we describe a modified one-step method for rapid extraction of reduced and oxidized CoQ followed by direct sample injection onto an HPLC column. Tandem HPLC runs of samples (±*p*-benzoquinone) furnish highly reproducible values for the oxidized, reduced, and total cofactor concentrations in mammalian cells and tissues. We demonstrate that the redox state of CoQ is sensitive to cell culture conditions, for example, hypoxia or chronic exposure to low H_2_S levels, *versus* normoxia, and undergoes a rapid oxidative shift when hypoxically grown cells are exposed to ambient air. Perturbations in the ETC by genetic or pharmacological means, or by dissipation of the mitochondrial but not the cytoplasmic NADH pool with *Lb*NOX (22), differentially shift the CoQ redox equilibrium. Our study provides insights into the sensitivity of the CoQ redox node to bioenergetic alterations, with implications for cellular antioxidant capacity.

## Results

### HPLC assay for CoQ analysis

**Figure 2:**
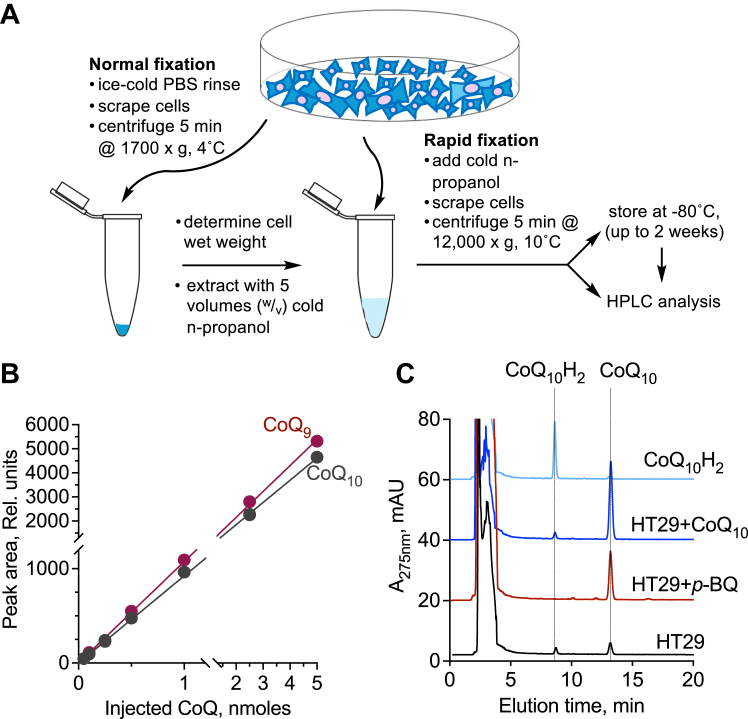
**HPLC analysis of CoQ**_**10**_**in samples.***A*, outline of cell sample preparation with (normal fixation) or without (rapid fixation) an intermediate step in which cells are scraped in PBS. *B*, calibration curves for CoQ_9_ and CoQ_10_ standards. *C*, HT29 cell samples exhibit two peaks assigned as CoQ_10_H_2_ and CoQ_10_ (*black*); oxidation with *p*-benzoquinone (*p*-BQ) leads to the disappearance of the CoQ_10_H_2_ and an increase in the intensity of CoQ_10_ peak (*red*). Spiking with authentic samples of CoQ_10_ (*dark blue*) or CoQ_10_H_2_ (*light blue*), confirmed the assignment of the corresponding peaks in the cell sample. The data are representative of at least three independent runs.

We optimized a simple and rapid one-step procedure for extracting CoQ_10_ from intact cells followed by direct injection into an HPLC column for estimating the concentration of the oxidized cofactor pool (Fig. 2*A*). While the use of *n*-propanol for CoQ extraction from food samples has been reported previously, the method involved drying and resuspension steps prior to HPLC analysis (16). In our method, conversion of CoQ_10_H_2_ to CoQ_10_ with *p*-benzoquinone (Fig. 1*A*) in samples followed by a second HPLC run, furnishes values for the total, and by subtraction, the reduced cofactor pool. The entire extraction and sample preparation method takes ∼15 min to complete and the sample can be processed immediately or stored at −80 °C without loss of signal intensity for at least 2 weeks. Oxidized CoQ_9_ and CoQ_10_ standards are well separated under our HPLC conditions and exhibit a linear dependence on the amount of injected sample between 0.05 to 5 nmols (Fig. 2*B*, r^2^ = 0.99).

Chromatograms of samples extracted from human cells showed two well-separated peaks, which were assigned as CoQ_10_H_2_ and CoQ_10_, respectively (Fig. 2*C*, *black trace*). Oxidation of the sample with *p*-benzoquinone resulted in the disappearance of the CoQ_10_H_2_ peak and a concomitant, albeit nonlinear, increase in the intensity of the CoQ_10_ (Fig. 2*C*, *red trace*). The difference in extinction coefficients between oxidized and reduced CoQ (Δε_ox-red(278 nm)_ = 12,000 M^−1^ cm^−1^) (23) explains the disproportionate increase in the CoQ_10_ peak intensity relative to the loss of CoQ_10_H_2_ intensity upon oxidation. Assignment of the oxidized and reduced cofactor peaks was confirmed by spiking the cell sample with authentic standards, which led to the expected increases in peak intensity (Fig. 2*C*, *light and dark blue traces*). Sample recovery was 107% (CoQ_10_H_2_) and 114% (CoQ_10_), similar to the 90 to 117% recovery reported in other studies (24, 25, 26, 27).

The concentrations of CoQ_10_H_2_ and CoQ_10_ were measured in three cell lines (Table 1). Interestingly, the total concentration as well as the CoQ_10_H_2_:CoQ_10_ ratio varied almost 2-fold between them. The human endothelial cell line EA.hy926 had approximately half the total cofactor levels and its redox state was more oxidized (CoQ_10_H_2_:CoQ_10_ = 1.7 ± 0.2) compared to the colon adenocarcinoma line, HT29 (2.3 ± 0.6). On the other hand, the total cofactor pool in osteosarcoma 143B cybrids was comparable to that of HT29 cells, but the cofactor pool was considerably more oxidized (1.2 ± 0.6).

**Table 1:** Concentration of oxidized, reduced, and total CoQ_10_ and CoQ_10_H_2_/CoQ_10_ redox ratio in human cell lines^a^

Cell line	CoQ_10_ (μmol/kg cells)	CoQ_10_H_2_ (μmol/kg cells)	Total (μmol/kg cells)	CoQ_10_H_2_/CoQ_10_
HT29	4.1 ± 0.5 (n = 8)	10.5 ± 0.4 (n = 8)	14.6 ± 0.5 (n = 8)	2.3 ± 0.6 (n = 23)
143B cybrid	7.0 ± 2.4 (n = 6)	7.1 ± 0.5 (n = 6)	14.1 ± 2.3 (n = 6)	1.2 ± 0.6 (n = 6)
EA.hy926	3.1 ± 0.2 (n = 4)	4.8 ± 0.8 (n = 4)	7.9 ± 0.9 (n = 4)	1.7 ± 0.2 (n = 8)

a The values represent the mean ± SD of the indicated number of independent experiments (n).

### Differential effects of ETC inhibitors on CoQ redox state

**Figure 3:**
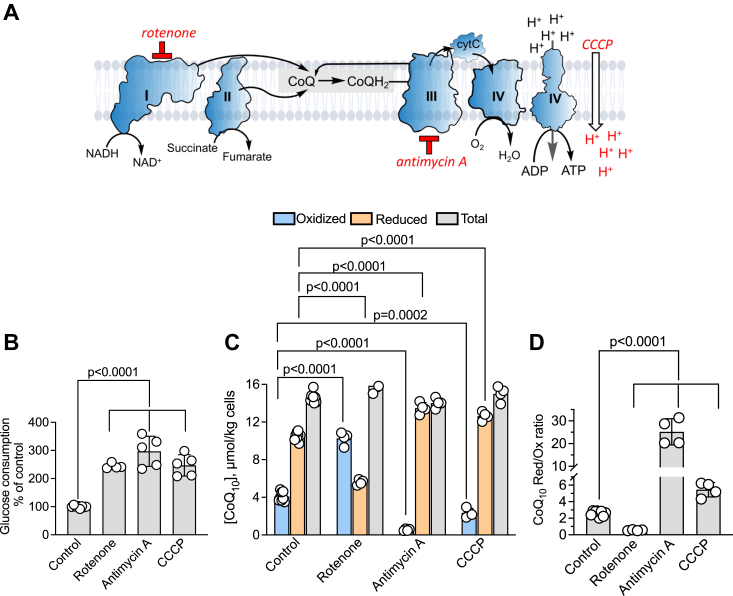
**ETC inhibitors differentially affect CoQ**_**10**_**redox status in human cells.***A*, scheme showing the targets of ETC inhibitors used in this study. *B* and *C*, treatment of HT29 cells with 5 μM rotenone (Rot), 100 nM antimycin A (AA), or 10 μM CCCP increased glucose consumption (*B*) and affected the relative concentrations of oxidized and reduced but not total CoQ_10_ levels (*C*) (n ≥ 4 independent experiments). *D*, the effect of ETC inhibitors in (*C*) on the ratio of reduced to oxidized CoQ_10_.

Inhibition of the ETC or oxidative phosphorylation decreases mitochondrial ATP production, and, depending on the locus of inhibitor action, is expected to differentially impact the CoQ redox state (Fig. 3*A*). Rotenone (complex I inhibitor), antimycin A (complex III inhibitor), and [(3-chlorophenyl)hydrazono]malononitrile (CCCP, uncoupler), each increased glucose consumption, consistent with activation of aerobic glycolysis (Fig. 3*B*), resulting from decreased ATP synthesis *via* oxidative phosphorylation. Rotenone decreased the CoQ_10_H_2_ pool and therefore, the reduced/oxidized CoQ ratio (Fig. 3, *C* and *D*), consistent with complex I being a major entry point for electrons into the ETC. On the other hand, antimycin A caused a significant reductive shift in the CoQ pool, while CCCP, which is expected to accelerate electron transfer by uncoupling it from proton transfer, also induced a reductive shift, albeit smaller (Fig. 3, *C* and *D*). While none of the treatments affected the total CoQ pool, the CoQ_10_H_2_:CoQ_10_ ratio changed from 2.3 ± 0.6 (untreated control) to 0.5 ± 0.05 (rotenone) to 25.1 ± 5.7 (antimycin A) to 5.5 ± 0.9 (CCCP) in response to the various inhibitors.

### Effects of ETC disruption on CoQ redox state

**Figure 4:**
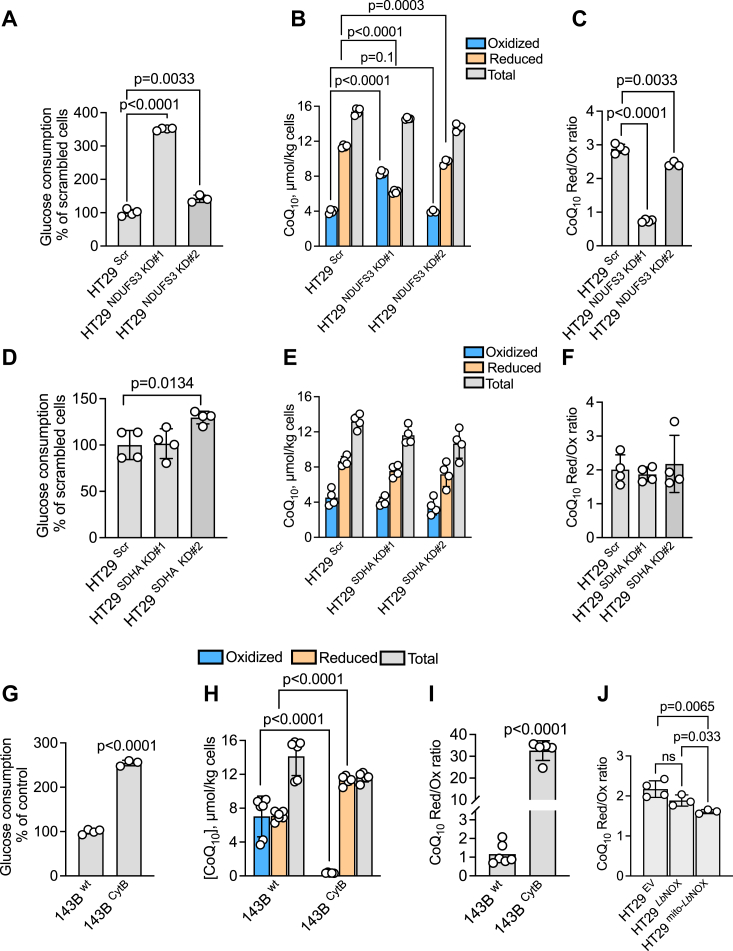
**Effects of ETC perturbations on CoQ redox state.***A*–*C*, knockdown of the mitochondrial complex I subunit NDUFS3 using two different shRNAs (#1 and 2), activated glucose consumption relative to scrambled control HT29 cells (*A*), affected the distribution of the oxidized and reduced CoQ_10_ pools (*B*), and the CoQ redox status (*C*) (n = 3 or 4). *D*–*F*, knockdown of the mitochondrial complex II subunit SDHA using two shRNAs (#1 and 2), did not have a sizeable impact on glucose consumption (*D*), or the distribution of the oxidized and reduced CoQ_10_ pools (*E*), and the CoQ redox status (*F*) (n = 4). *G*–*I*, 143B CytB cybrids showed increased glucose consumption (*G*), decreased total CoQ_10_ levels (*H*), and a large reductive shift in the CoQ_10_ pool (*I*) relative to wild-type 143B cybrids (n = 4–6). *J*, HT29 cells expressing *Lb*NOX in the mitochondrion but not in the cytosol exhibited an oxidative shift in the CoQ_10_ pool.

The effect of ETC dysfunction was also investigated by genetic ablation of complexes I-III. Knockdown of the mitochondrial complex I NDUFS3 subunit, phenocopied the rotenone effect by increasing glucose consumption and decreasing CoQ_10_H_2_ levels without affecting the total pool (Fig. 4, *A*–*C*). Interestingly, the knockdown efficiency using two different shRNAs resulted in either undetectable (KD#1) or a 70% decrease (KD#2) in NDUFS3 expression (6). The differences in NDUFS3 knockdown efficiency were correlated with a 250% (KD#1) and 40% (KD#2) increase in glucose consumption compared to the scrambled control (Fig. 4*A*). Similarly, differences in the magnitude of the oxidative shift in the CoQ pool were observed in the two NDUFS3 knockdown lines (Fig. 4, *B* and *C*).

The two shRNA sequences used to target the complex II SDHA subunit resulted in a comparable decrease in SDHA protein levels (∼75 and 88%) as reported previously (6). The glucose consumption rates of the two SDHA knockdown lines were more or less comparable to scrambled controls and significant changes in CoQ pool size or redox state were not observed (Fig. 4, *D*–*F*). These data indicate that the contribution of complex II to electron flux in the ETC is low under our experimental conditions.

The 143B CytB cybrid lacks a functional complex III (28) and exhibits increased glucose consumption and a pronounced reductive shift in the CoQ pool compared to the wild-type 143B cybrid (Fig. 4, *G*–*I*). In fact, the redox state of the CoQ pool in the 143B CytB cybrid was comparable to HT29 cells treated with antimycin A, revealing the profound effect of complex III inhibition on the availability of oxidized CoQ. The CoQ_10_H_2_:CoQ_10_ ratio increased from 1.2 ± 0.6 to 32.6 ± 4.5 in wild-type *versus* 143B CytB cybrids (Fig. 4*I*). Finally, dissipation of the NADH pool by expression of the H_2_O-generating NADH oxidase, *Lb*NOX, elicited a modest oxidative shift in the CoQ pool, albeit only when it was expressed in the mitochondrion but not in the cytoplasm (Fig. 4*J*). The CoQ_10_H_2_:CoQ_10_ ratio decreased from 2.2 ± 0.2 in HT29 cells expressing the empty vector control to 1.6 ± 0.5 in cells expressing *Lb*NOX in the mitochondrion.

### Hypoxia causes a reductive shift in the CoQ pool

**Figure 5:**
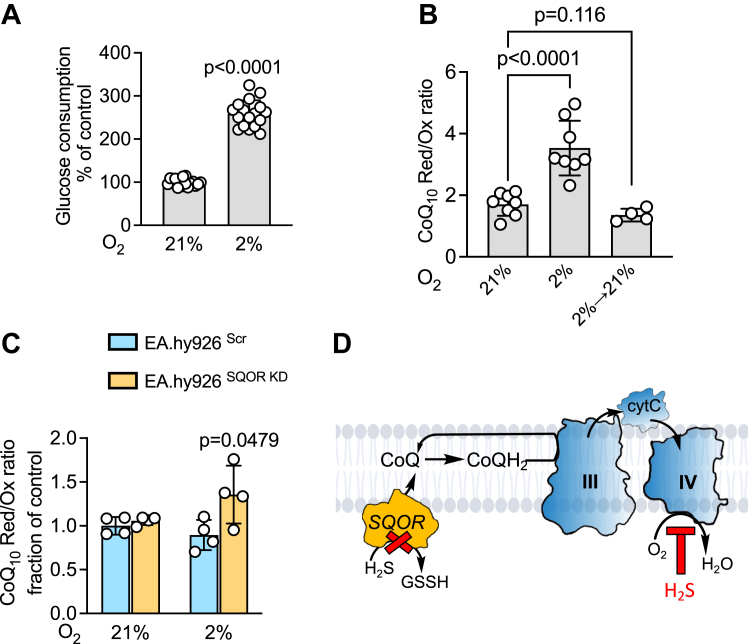
**Dependence of the effect of hypoxia on the CoQ**_**10**_**poolincelllines.***A* and *B*, glucose consumption increased in HT29 cells cultured in 2% (hypoxia) *versus* 21% (normoxia) O_2_ (*A*, n= 16 or 17) and is associated with a reductive shift in the CoQ_10_ pool, which was reversed to control levels within 10 min of exposure of cells to ambient air (*B*, n= 4–8). *C* and *D*, hypoxia did not affect the CoQ_10_ redox status in scrambled control EA.hy926 cells but caused a reductive shift when SQOR was knocked down (*C*, n = 4), which is predicted to increase H_2_S level (D).

Glucose consumption is activated when cells are cultured under hypoxic (2% O_2_) *versus* normoxic (21% O_2_) conditions (Fig. 5*A*). While reduced and oxidized CoQ is relatively stable following extraction into *n*-propanol, we found that the CoQ_10_H_2_ pool shifted rapidly when hypoxically grown cells were moved to ambient air (Fig. 5*B*). Thus, within 10 min of exposure of HT29 cells to normoxia, the effect of hypoxic culture on the intracellular CoQ redox poise was reversed and the CoQ_10_H_2_:CoQ_10_ ratio returned to normoxic levels. Rapid fixation by the addition of *n*-propanol directly to the culture dish (Fig. 2*A*) was therefore necessary to minimize perturbations in the CoQ redox state during sample preparation. It is important to note that while the rapid fixation method allows assessment of the CoQ redox state, information about CoQ concentration cannot be readily obtained from these samples since the weight of the cell pellet is not determined.

In endothelial cells, glycolysis is estimated to support up to 85% of the energy demand (29). Consistent with the predominance of glycolysis to fuel energy needs, EA.hy926 cells do not exhibit a change in the CoQ redox status when cultured in 2% *versus* 21% O_2_ (Fig. 5*C*). However, in contrast to scrambled controls, EA.hy926 cells harboring an SQOR knockdown showed a 1.5-fold increase in the CoQ_10_H_2_:CoQ_10_ ratio. Hypoxia increases H_2_S biogenesis in EA.hy926 cells (30), which is predicted to accumulate under conditions of SQOR deficiency, inhibiting complex IV (Fig. 5*D*) and causing a reductive shift in the CoQ pool. The direct effect of sulfide on the CoQ redox state was tested next as discussed below.

### Acute and chronic sulfide exposure induces a reductive shift in the CoQ pool

**Figure 6:**
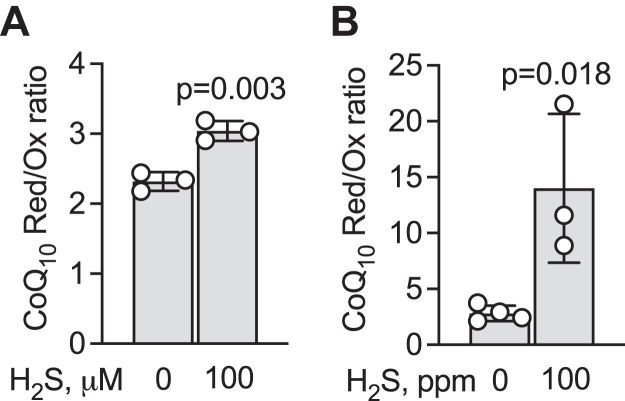
**Sulfide induces a reductive shift in HT29 cells.***A*, acute exposure to 100 μM Na_2_S for 4 h resulted in a small but statistically significant reductive shift in the CoQ pool (n = 3). *B*, chronic exposure of HT29 cells to 100 ppm H_2_S for 24 h led to a marked reductive shift in the CoQ pool (n = 3 or 4).

Sulfide is a well-known respiratory poison that targets the metal centers in complex IV (Fig. 5*D*) (31) and activates aerobic glycolysis across various cell lines (32, 33, 34). Despite its volatility, acute exposure of HT29 cells to sulfide (100 μM H_2_S bolus) leads to prolonged fractional inhibition of complex IV that is observable 4 h later (35). Under these conditions, a small (30%) but statistically significant reductive shift in the CoQ pool is observed (Fig. 6*A*). Chronic H_2_S exposure (100 ppm H_2_S, 24 h) leads to ∼20 μM dissolved sulfide in the culture medium, which results in complete inhibition of oxidative phosphorylation and an ∼4-fold activation of aerobic glycolysis in HT29 cells (36). Under these conditions, a 5-fold increase in CoQ_10_H_2_:CoQ_10_ ratio to 14.0 ± 6.6 was observed (Fig. 6*B*), which is substantially larger than seen under hypoxia (Fig. 5B).

### CoQ pool size and redox state in murine tissues

**Figure 7:**
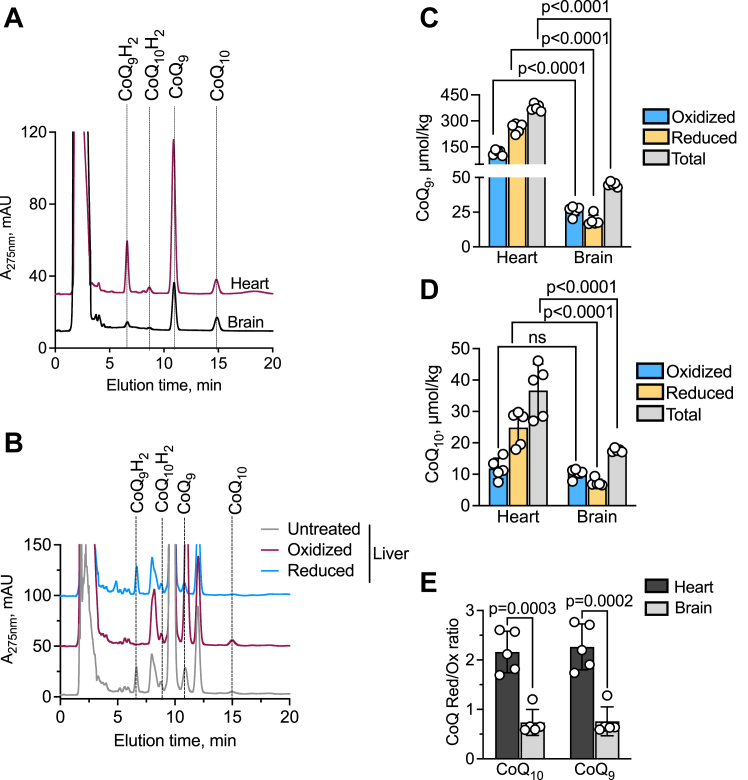
**Analysis of CoQ**_**9**_**and CoQ**_**10**_**pools in murine tissue.***A* and *B*, representative HPLC traces for CoQ analysis from murine heart and brain (*A*) and liver (*B*). In the liver, the presence of another substance that co-migrated with CoQ_9_ and CoQ_10_H_2_ led to residual peak intensity following reduction and oxidation, respectively and precluded quantitative analysis. *C* and *D*, concentrations of oxidized, reduced, and total CoQ_9_ (*C*) CoQ_10_ (*D*) in murine heart and brain. *E*, redox ratio of the two CoQ pools. n = 5 in all experiments.

The utility of our method for measuring CoQ levels was assessed with murine liver, heart, and brain tissue where CoQ_9_ is the major cofactor form (25). While the CoQ_9_ and CoQ_10_ peaks were well separated in brain and heart samples (Fig. 7*A*), they overlapped with other materials in liver samples, leading to residual absorbance in the CoQ_9_ peak following reduction, and in the CoQ_10_H_2_ peak following oxidation (Fig. 7*B*). The concentration of the CoQ pools in the liver could therefore not be reliably assessed under these conditions. The total CoQ_9_ concentration was 10- and 2.5-fold higher than CoQ_10_ in the heart and brain, respectively (Fig. 7*C*, D). Furthermore, the total CoQ_9_ and CoQ_10_ concentrations in the heart were 8- and 2-fold higher than in the brain (Fig. 7, *C* and *D*) as reported previously in rat and murine tissue (14, 25). Both CoQ_9_ and CoQ_10_ pools were more oxidized in the brain compared to the heart (Fig. 7*E*), as reported previously (14, 25).

## Discussion

Large tissue-dependent variations in CoQ levels and redox state have been reported, which are further influenced by development, as well as by other physiological and pathological factors (37, 38, 39). Our optimized one-step CoQ sample preparation for UV-detected HPLC analysis provides a convenient method for assessing the concentration and redox poise of CoQ_9_ and CoQ_10_ in mammalian cells and tissues. The method also allows monitoring of the CoQ redox status in response to genetic, pharmacological, and environmental (*e.g.*, O_2_, H_2_S, nutrient) perturbations. We observed a CoQ_10_H_2_:CoQ_10_ ratio ranging from 1.2 to 2.3 across three human cell lines (Table 1). While a more oxidized CoQ pool (0.54) was recently reported in cell culture (20), it is presently not known whether the value lies within the normal range for cell lines or results from more extensive sample handling.

It was previously reported that the redox state and size of the CoQ pool in samples prepared by rapid extraction into a hydrophobic solvent was stable during 24 h storage at 4 °C or −18 °C (14). We also found that the CoQ pool size and redox state following extraction into *n*-propanol were stable at −80 °C for 2 weeks, which was the longest duration over which stability was monitored in our study. In contrast to the relative stability of extracted samples, the intracellular redox state of CoQ revealed susceptibility to rapid changes as evidenced by a sizeable oxidative shift when hypoxically grown cells were exposed to ambient air for 10 min (Fig. 5*B*). Thus, when examining the effects of environmental triggers like H_2_S or a change in O_2_ exposure, it is important to use the rapid fixation method for extracting CoQ to preserve the redox poise (Fig. 2*A*).

In HT29 cells, which were characterized more extensively in our study, changes in the ETC flux induced by genetic or pharmacological means, led to redox shifts in the CoQ pool that could be explained by the locus of perturbation. With tissue samples, we found differences in the ratio of CoQ_9_ to CoQ_10_ between murine heart *versus* brain, and both pools were more reduced in heart. A highly reduced pool also reflects a higher antioxidant capacity and, in concert with the proton motive force and availability of terminal electron acceptors, can impact the direction of electron flow through complex III *versus* II or through complex I *via* reverse electron transfer (4).

Interestingly, the reductive shift in the CoQ pool that is elicited by chronic low-level sulfide exposure was substantially larger than that triggered by hypoxia, revealing the potential for sulfide to synergize with low O_2_ to modulate ETC flux as predicted by *P*_50O2_ measurements (35).

### Limitations of our study

CoQ is not uniformly distributed across membranes and while our study provides information on bulk concentrations of reduced and oxidized CoQ, it does not provide insights into local changes in organellar endo-membranes and the plasma membrane. Similarly, redox shifts in response to ETC perturbations, are likely to underestimate the redox shifts occurring in the mitochondrial membrane.

## Experimental procedures

### Reagents

Hexane (HPLC grade), isopropanol, methanol (HPLC grade), n-propanol, p-benzoquinone, CoQ_10_, CoQ_9_, NaBH_4_, antimycin A, [(3-chlorophenyl) hydrazono] malononitrile (CCCP), Na_2_S, rotenone, were purchased from Sigma. Cell culture reagents (cell culture media, FBS, Pen/Strep) and geneticin were from Gibco. Puromycin and doxycycline were purchased from Sigma.

### Mice

Female C57BL mice were purchased from the Jackson Laboratory (Bar Harbor, ME, USA) and maintained under standard housing conditions with ad libitum access to food and water and 12 h light-day cycle. For organ collection, 2-month-old female mice were euthanized with CO_2_ using a procedure approved by the University of Michigan Committee on the Use and Care of Animals, which is based on the University of Michigan Laboratory Animal Medicine guidelines. Liver, heart, and brain were harvested quickly, frozen immediately in liquid nitrogen, and stored at −80 °C until use.

### Cell culture conditions

Human colorectal adenocarcinoma HT29 cells and human somatic hybrid cells EA.hy926, were obtained from American Type Culture Collection. HT29 cells expressing the bacterial water-producing NADH oxidase, *Lb*NOX were prepared as described previously (32). HT29 cells in which the complex I subunit NDUFS3 was knocked down with two shRNAs were prepared as described elsewhere (6). Human osteosarcoma 143B^WT^ and 143B^Cytb^ cybrids (28) were a generous gift from Dr Matthew Vander Heiden (MIT). HT29 cells were cultured in RPMI 1640 medium containing 25 mM HEPES and supplemented with 10% FBS, 100 units/ml penicillin, and 100 μg/ml streptomycin. The same medium was also used to culture scrambled and NDUFS3 KD HT29 cells but with the addition of 1 μg/ml puromycin. 143B^WT^ and 143B^Cytb^ cybrids were cultured in DMEM supplemented with 10% FBS, 100 units/ml penicillin, 100 μg/ml streptomycin, and 0.1 mg/ml uridine. EA.hy926 cells (scrambled and SQOR KD) were prepared as described previously (30), cultured in DMEM medium supplemented with 10% FBS, 100 units/ml penicillin, 100 μg/ml streptomycin and 1 μg/ml puromycin.

HT29 cells containing an empty vector (EV) or *Lb*NOX (cytoplasmic or mitochondria version) were cultured in RPMI 1640 medium containing 25 mM HEPES and supplemented with 10% FBS, 100 units/ml penicillin, and 100 μg/ml streptomycin supplemented with 300 μg/ml geneticin. To induce *Lb*NOX expression, cells were cultured in the presence of doxycycline (300 ng/ml) for 24 h. The same concentration of doxycycline was also added to cells expressing the empty vector. All cells were maintained at 37 °C in a cell culture incubator with a humidified atmosphere of ambient air (21% O_2_), supplemented with 5% CO_2_. Alternatively, cells were cultured in a hypoxia incubator with a humidified atmosphere containing 93% N_2_, 2% O_2_, and 5% CO_2_.

### CoQ_10_ and glucose consumption during ETC inhibition under normoxia

HT29 cells were cultured in 10 cm plates to a confluency of 70 to 80%. The medium was changed (20 ml/plate) and antimycin A (1 μl/ml of a 100 μM stock solution in ethanol, 100 nM final concentration), or CCCP (2.5 μl/ml of a 4 mM stock solution in ethanol, 10 μM final concentration), or rotenone (1 μl/ml of a 5 mM stock solution in DMSO, 5 μM final concentration) was added. The corresponding volume of vehicle (ethanol or DMSO) was added to control samples in experiments in which antimycin A, CCCP, or rotenone was used. Cells were cultured for 15 h following the addition of the inhibitor; samples for glucose analysis were collected at time = 0 and 15 h. Samples for CoQ_10_ analysis were collected after 15 h culture.

HT29 cells (scrambled, and NDUFS3 KD) and cybrids (143B^WT^ and 143B^Cytb^) were grown in 10 cm plates. At the beginning of the experiment, the medium was changed (20 ml/plate) and cells were cultured under normoxic conditions for 9 h (HT29 cells) or 15 h (143B^WT^ and 143B^Cytb^ cybrids). Aliquots for glucose analysis were collected at the beginning and end of the experiment with HT29 cells. Samples for CoQ_10_ analysis in the cells were collected at the end of the 9 or 15 h incubation.

*Lb*NOX or empty vector-expressing HT29 cells were grown in 6 cm plates to 70 to 80% confluency. Then, the culture medium was changed (6 ml/plate) and doxycycline was added to a final concentration of 300 ng/ml to induce *Lb*NOX expression. After 24 h, the medium was replaced with fresh medium (4 ml/plate) and incubated for 5 h after which CoQ_10_ samples were extracted for analysis.

### CoQ_10_ and glucose consumption

HT29 cells were cultured to confluency in 6- or 10-cm plates and, following a medium change, the plates were moved to normoxic (21%) or hypoxic (2% O_2_) incubators for 9 h. Samples for CoQ_10_ analysis were prepared using the fast fixation protocol while aliquots for glucose analysis were removed at t = 0 or 9 h. To assess CoQ_10_ recovery after hypoxia, the medium was aspirated after 9 h and replaced with a 2 ml cold PBS/6 cm plate, which was moved to the laboratory bench (ambient air and temperature) for 10 min after which samples were collected using the “fast fixation” protocol.

EA.hy926 cells (scrambled and SQOR KD) were seeded at a density of 3 × 10^6^ in a 10 ml medium/10 cm plate. The next day, the medium was changed (10 ml/plate), and cells were placed in normoxic or hypoxic incubator for 24 h and harvested for CoQ_10_ quantitation.

### Effect of H_2_S on CoQ_10_ redox status

The effect of acute sulfide treatment was studied by seeding 5 × 10^6^ HT29 cells in 10 cm plates (10 ml medium) and cultured for 3 days with a medium change after day 2. On day 3, cells were at ∼70% confluency and the medium was changed again and a freshly prepared solution of Na_2_S in water was added to a final concentration of 100 μM. Samples were harvested after 4 h for CoQ_10_ quantitation.

The effect of chronic sulfide treatment was studied by seeding 7.2 × 10^6^ HT29 cells in 10 cm plate (10 ml medium/plate) and cultured overnight in in a normoxic incubator. The next day, the medium was changed (20 ml medium/plate) and cultures were placed in a sulfide growth chamber (36) with 100 ppm H_2_S. Control cells were cultured under the same conditions (*i.e.*, humidified air containing 5% CO_2_ but lacking H_2_S). Aliquots of the culture medium were removed at t = 0 and 24 h for glucose analysis and samples for CoQ_10_ quantitation were prepared at 24 h.

### Glucose analysis

For glucose analysis, 50 μl aliquot of RPMI 1640 cell culture medium was mixed with 100 μl 5% HClO_4_, or 30 μl DMEM medium with 150 μl 5% HClO_4_. Samples were mixed by vortexing, centrifuged (5 min, 10,000*g*, 4 °C), and the supernatant was aspirated, neutralized to pH ∼7.0 with a saturated K_2_CO_3_ solution, and stored at −20 °C until use. Glucose concentration was measured using a D-GLUCOSE-HK kit (Megazyme) according to the manufacturer’s protocol.

### Sample preparation for the CoQ analysis

For CoQ analysis in mammalian cells and tissues, we used a modification of the HPLC-based protocols reported previously (17, 24, 25, 40, 41). Briefly, for “normal fixation” of cell samples, the culture plate was placed on ice, the medium was aspirated, and ice-cold PBS was added (1 ml/10 cm plate or 0.5 ml/6 cm plate). Then, cells were scraped, and the suspension was transferred to a pre-weighed 1.5 ml Eppendorf tube and centrifuged at 1700*g* for 5 min at 4 °C. The supernatant was aspirated and the wet weight of the cell pellet was determined. The pellet was rapidly mixed with 5 volumes of n-propanol (^w^/_v_) to avoid formation of aggregates. The sample was allowed to stand at room temperature for 1 to 2 min, vortexed, and centrifuged at 12,000*g* for 5 min at 10 °C to avoid precipitation of CoQ_10_ in n-propanol. Following centrifugation, samples were kept on ice, the supernatant was collected and stored at −80 °C. The CoQ_10_ pool size and redox states were stable for at least 2 weeks under these conditions.

We also developed a “rapid fixation” protocol to extract CoQ_10_ from cells more quickly. For this, the culture plate was placed on ice, the medium was aspirated and ice-cold n-propanol was added to the plate (1 ml/10 cm plate or 0.5 ml/6 cm plate) and the plate was gently rocked so that the cells were covered with propanol. Cells were scraped and the suspension was transferred to a 1.5 ml tube, which was allowed to stand at room temperature for 2 min and processed as described above. The time between moving a culture plate from an incubator to covering cells with n-propanol, which denatures cells, was <1 min. To adjust for the lower concentration of CoQ_10_ in EA.hy926 cells, frozen samples were thawed, concentrated 3-fold using a SpeedVac, and stored at −80 °C. The rapid fixation method was used in experiments where the effects of hypoxia, H_2_S and *Lb*NOX expression were studied and allowed estimation of the reduced:oxidized CoQ_10_ ratio only. In all other experiments, the normal fixation protocol was used and allowed estimation of the reduced and oxidized cofactor levels in addition to estimation of the redox state.

Frozen mouse tissue samples were homogenized on ice using four volumes of n-propanol (^w^/_v_) for the liver and brain and in six volumes of n-propanol (^w^/_v_) for heart, using a glass homogenizer. The homogenates were centrifugated at 12,000*g* for 5 min at 10 °C, the supernatants were collected, and stored at −80 °C until use.

### HPLC analysis

HPLC analysis of CoQ was performed on a Hypersil ODS column (150 × 4.6 mm, 3 μ bead size, Thermo Fisher), or Microsorb-MV 100-5 C18 column (150 × 4.6 mm, 5 μ bead size, Agilent). Similar results were obtained with both columns. Samples were eluted isocratically at room temperature with a flow rate of 0.8 ml/min using a solvent, comprising isopropanol (15 ml), methanol (845 ml), and hexane (140 ml) in a total volume of 1 l. Peaks were detected by UV absorbance at 275 nm. Oxidized and reduced CoQ exhibit absorbance maxima at 278 nm (ε = 14,500 M^−1^ cm^−1^) and 287 nm (ε = 3340 M^−1^ cm^−1^) (23), respectively, and calibration curves were generated with oxidized CoQ_9_ and CoQ_10_ samples of known concentration prepared in *n*-propanol.

Samples stored at −80 °C were thawed and injected into the column. Each sample was run twice, before and after oxidation of reduced CoQ with *p*-benzoquinone. For this, 115 μl of the sample was mixed with 5 μl of *p*-benzoquinone (4 mg/ml in water), and the mixture was kept for 5 min at +10 °C in a temperature-controlled autosampler tray before being injected into the column. The first run provided the concentration of oxidized CoQ while the second run furnished information on the total CoQ pool allowing estimation of the reduced CoQ (CoQH_2_) pool. To validate the assigned CoQ_10_H_2_ peak, CoQ_10_ (115 μl of 1 mM in n-propanol) was reduced with NaBH_4_ (5 μl of a 100 mM stock solution in water) for 5 min at 25 °C. The reduction was confirmed by the conversion of the 278 nm CoQ_10_ peak to the 287 nm CoQ_10_H_2_ peak, which was spiked into the cell or tissue lysate. Cellular and tissue CoQ_n_/CoQ_n_H_2_ concentrations were estimated by taking into account the sample wet weight and dilutions during sample preparation. Concentrations were expressed in units of μmol/kg wet weight, which is roughly equivalent to micromolar CoQ_n_/CoQ_n_H_2_.

### Statistical analysis

The student’s *t* test (two-sided) was used to obtain *p* values using GraphPad Prism. Exact values are reported to three decimal places for significant differences (<0.05). Differences below 0.0001 are reported as <0.0001.

## Data availability

All data are contained within the manuscript.

## Conflict of interest

The authors declare that they have no conflicts of interest with the contents of this article.

## Footnotes

‡ CoQ is used generically to refer to the cofactor without specifying the redox state or the isoprenoid tail length.

## References

[1] Crane F.L., Hatefi Y., Lester R.L., Widmer C. (1957). Isolation of a quinone from beef heart mitochondria. Biochim. Biophys. Acta.

[2] Morton R.A. (1958). Ubiquinone. Nature.

[3] Wang Y., Hekimi S. (2016). Understanding ubiquinone. Trends Cell Biol..

[4] Banerjee R., Kumar R. (2022). Gas regulation of complex II reversal *via* electron shunting to fumarate in the mammalian ETC. Trends Biochem. Sci..

[5] Chouchani E.T., Pell V.R., Gaude E., Aksentijevic D., Sundier S.Y., Robb E.L. (2014). Ischaemic accumulation of succinate controls reperfusion injury through mitochondrial ROS. Nature.

[6] Kumar R., Landry A.P., Guha A., Vitvitsky V., Lee H.J., Seike K. (2022). A redox cycle with complex II prioritizes sulfide quinone oxidoreductase-dependent H_2_S oxidation. J. Biol. Chem..

[7] Spinelli J.B., Rosen P.C., Sprenger H.G., Puszynska A.M., Mann J.L., Roessler J.M. (2021). Fumarate is a terminal electron acceptor in the mammalian electron transport chain. Science.

[8] Finkel T. (2011). Signal transduction by reactive oxygen species. J. Cell Biol..

[9] Seshadri Sastry P., Jayaraman J., Ramasarma T. (1961). Distribution of coenzyme Q in rat liver cell fractions. Nature.

[10] Bersuker K., Hendricks J.M., Li Z., Magtanong L., Ford B., Tang P.H. (2019). The CoQ oxidoreductase FSP1 acts parallel to GPX4 to inhibit ferroptosis. Nature.

[11] Stefely J.A., Pagliarini D.J. (2017). Biochemistry of mitochondrial coenzyme Q biosynthesis. Trends Biochem. Sci..

[12] Franza T., Gaudu P. (2022). Quinones: more than electron shuttles. Res. Microbiol..

[13] Anand A., Chen K., Yang L., Sastry A.V., Olson C.A., Poudel S. (2019). Adaptive evolution reveals a tradeoff between growth rate and oxidative stress during naphthoquinone-based aerobic respiration. Proc. Natl. Acad. Sci. U. S. A..

[14] Aberg F., Appelkvist E.L., Dallner G., Ernster L. (1992). Distribution and redox state of ubiquinones in rat and human tissues. Arch. Biochem. Biophys..

[15] Yuan B., Liu C., Xu P., Lin L., Pan C., Wang L. (2011). Validated HPLC method for the quantitative determination of CoQ(10) in dog plasma and its application to a pharmacokinetic study. Biomed. Chromatogr..

[16] Semeniuc C.A., Ranga F., Podar A.S., Ionescu S.R., Socaciu M.I., Fogarasi M. (2023). Determination of coenzyme Q10 content in food by-products and waste by high-performance liquid chromatography coupled with diode array detection. Foods.

[17] Barshop B.A., Gangoiti J.A. (2007). Analysis of coenzyme Q in human blood and tissues. Mitochondrion.

[18] Tang P.H., Miles M.V., DeGrauw A., Hershey A., Pesce A. (2001). HPLC analysis of reduced and oxidized coenzyme Q(10) in human plasma. Clin. Chem..

[19] Pallotti F., Bergamini C., Lamperti C., Fato R. (2021). The roles of coenzyme Q in disease: direct and indirect involvement in cellular functions. Int. J. Mol. Sci..

[20] Burger N., Logan A., Prime T.A., Mottahedin A., Caldwell S.T., Krieg T. (2020). A sensitive mass spectrometric assay for mitochondrial CoQ pool redox state *in vivo*. Free Radic. Biol. Med..

[21] Pandey R., Riley C.L., Mills E.M., Tiziani S. (2018). Highly sensitive and selective determination of redox states of coenzymes Q(9) and Q(10) in mice tissues: application of orbitrap mass spectrometry. Anal. Chim. Acta.

[22] Titov D.V., Cracan V., Goodman R.P., Peng J., Grabarek Z., Mootha V.K. (2016). Complementation of mitochondrial electron transport chain by manipulation of the NAD^+^/NADH ratio. Science.

[23] Jackson M.R., Melideo S.L., Jorns M.S. (2012). Human sulfide:quinone oxidoreductase catalyzes the first step in hydrogen sulfide metabolism and produces a sulfane sulfur metabolite. Biochemistry.

[24] Mosca F., Fattorini D., Bompadre S., Littarru G.P. (2002). Assay of coenzyme Q(10) in plasma by a single dilution step. Anal. Biochem..

[25] Tang P.H., Miles M.V., Miles L., Quinlan J., Wong B., Wenisch A. (2004). Measurement of reduced and oxidized coenzyme Q9 and coenzyme Q10 levels in mouse tissues by HPLC with coulometric detection. Clin. Chim. Acta.

[26] Tang P.H., Miles M.V., Steele P., Davidson B.S., Geraghty S.R., Morrow A.L. (2006). Determination of coenzyme Q10 in human breast milk by high-performance liquid chromatography. Biomed. Chromatogr..

[27] Edlund P.O. (1988). Determination of coenzyme Q10, alpha-tocopherol and cholesterol in biological samples by coupled-column liquid chromatography with coulometric and ultraviolet detection. J. Chromatogr..

[28] Sullivan L.B., Gui D.Y., Hosios A.M., Bush L.N., Freinkman E., Vander Heiden M.G. (2015). Supporting aspartate biosynthesis is an essential function of respiration in proliferating cells. Cell.

[29] De Bock K., Georgiadou M., Schoors S., Kuchnio A., Wong B.W., Cantelmo A.R. (2013). Role of PFKFB3-driven glycolysis in vessel sprouting. Cell.

[30] Kumar R., Vitvitsky V., Sethaudom A., Singhal R., Solanki S., Alibeckoff s. (2024). Sulfide oxidation promotes hypoxic angiogenesis and neovascularization. Nat. Chem. Biol..

[31] Cooper C.E., Brown G.C. (2008). The inhibition of mitochondrial cytochrome oxidase by the gases carbon monoxide, nitric oxide, hydrogen cyanide and hydrogen sulfide: chemical mechanism and physiological significance. J. Bioenerg. Biomembr..

[32] Vitvitsky V., Kumar R., Libiad M., Maebius A., Landry A., Banerjee R. (2021). The mitochondrial NADH pool is involved in hydrogen sulfide signaling and stimulation of aerobic glycolysis. J. Biol. Chem..

[33] Hanna D., Kumar R., Banerjee R. (2023). A metabolic paradigm for hydrogen sulfide signaling *via* electron transport chain plasticity. Antioxid. Redox Signal..

[34] Kumar R., Banerjee R. (2021). Regulation of the redox metabolome and thiol proteome by hydrogen sulfide. Crit. Rev. Biochem. Mol. Biol..

[35] Hanna D.A., Diessl J., Guha A., Kumar R., Andren A., Lyssiotis C. (2024). H(2)S preconditioning induces long-lived perturbations in O(2) metabolism. Proc. Natl. Acad. Sci. U. S. A..

[36] Hanna D.A., Vitvitsky V., Banerjee R. (2023). A growth chamber for chronic exposure of mammalian cells to H(2)S. Anal. Biochem..

[37] Kalen A., Appelkvist E.L., Dallner G. (1989). Age-related changes in the lipid compositions of rat and human tissues. Lipids.

[38] Matsura T., Yamada K., Kawasaki T. (1991). Changes in the content and intracellular distribution of coenzyme Q homologs in rabbit liver during growth. Biochim. Biophys. Acta.

[39] Huertas J.R., Battino M., Lenaz G., Mataix F.J. (1991). Changes in mitochondrial and microsomal rat liver coenzyme Q9 and Q10 content induced by dietary fat and endogenous lipid peroxidation. FEBS Lett..

[40] Littarru G.P., Mosca F., Fattorini D., Bompadre S., Battino M. (2004). Assay of coenzyme Q10 in plasma by a single dilution step. Methods Enzymol..

[41] Robb E.L., Hall A.R., Prime T.A., Eaton S., Szibor M., Viscomi C. (2018). Control of mitochondrial superoxide production by reverse electron transport at complex I. J. Biol. Chem..

